# COVID-19 vaccine uptake in Skåne county, Sweden, in relation to individual-level and area-level sociodemographic factors: a register-based cross-sectional analysis

**DOI:** 10.1136/bmjph-2023-000437

**Published:** 2024-03-25

**Authors:** Adam Mitchell, Malin Inghammar, Louise Bennet, Per-Olof Östergren, Mahnaz Moghaddassi, Jonas Björk

**Affiliations:** 1 Department of Occupational and Environmental Medicine, Lund University, Lund, Sweden; 2 Department of Clinical Sciences Lund, Lund University, Lund, Sweden; 3 Department of Infectious Diseases, Skåne University Hospital Lund, Lund, Sweden; 4 Skåne University Hospital, Lund, Sweden; 5 Department of Clinical Sciences Malmö, Family Medicine, Lund University, Lund, Sweden; 6 Social Medicine and Global Health, Department of Clinical Sciences Malmö, Lund University, Malmö, Sweden

**Keywords:** COVID-19, Epidemiology, Public Health, Cross-Sectional Studies, Confounding Factors, Epidemiologic

## Abstract

**Objectives:**

Better understanding of societal factors associated with COVID-19 vaccination can have important implications for public health policy to increase uptake.

**Methods:**

This study investigated sociodemographic determinants of COVID-19 vaccine uptake with ≥2 doses vs 0 doses, and ≥3 doses vs 2 doses, among adults (≥18 years) in a general population from Sweden followed from 27 December 2020 (n=1 064 548 at the present cross-section—12 June 12 2022). Associations between individual-level and area-level sociodemographic factors and vaccine uptake were modelled with logistic regression, with average marginal effects and estimated proportion vaccinated subsequently estimated.

**Results:**

Being vaccinated with ≥2 doses vs 0 doses was positively associated with education (tertiary vs primary, OR 1.5, 95% CI 1.3 to 1.7), household disposable income (Q5 vs Q1, OR 2.3; 95% CI 1.9 to 2.7), comorbidities (≥2 doses vs none, OR 1.9, 95% CI 1.8 to 1.9) and residential area type (affluent socioeconomic conditions vs poor, OR 2.0, 95% CI 1.6 to 2.4). Whereas, being born outside Sweden was associated with a lower uptake (low and middle-income countries vs Swedish born, OR 0.6, 95% CI 0.5 to 0.7). The associations were generally similar when comparing booster vs remaining on only two doses. From these ORs, there were consistent differences in the estimated proportion vaccinated both for ≥2 doses and booster vaccination. Absolute changes in percentage vaccinated between affluent and poor areas were largely similar across individual country of birth, income and education, both for at least two doses and for the booster doses.

**Conclusions:**

COVID-19 vaccine uptake was associated with higher sociodemographic classifications both at the individual level and area level. The predicted proportion vaccinated increased with more affluent socioeconomic conditions and concurrent increases in individual household income were the strongest indicators. This sociodemographic selection showed consistency with respect to entering (obtaining ≥2 doses) and remaining (obtaining at least one booster dose) in the vaccination programme.

**WHAT IS ALREADY KNOWN ON THIS TOPIC**

**WHAT THIS STUDY ADDS**

- What we now know as a result of this study is that differences in individual and societal sociodemographic determinants of COVID-19 vaccination are consistent not only when comparing two or more doses with 0 doses but also for further booster vaccination. Previous work has reported on differences in individual and area-wide sociodemographic determinants of vaccination uptake yet have not been able to report on the combination in the same individuals. This study also adds evidence that individual household income and area-level socioeconomic condition are more strongly associated with vaccine uptake than education.

**HOW THIS STUDY MIGHT AFFECT RESEARCH, PRACTICE OR POLICY**

- In an ideal system, vaccination should be equally informed free choice but our results show that this is not the case. Sex, age, education, household income, area socioeconomic condition and place of birth were all strongly associated with either greater or lower odds of vaccination. This suggests that specific groups and specific areas with socioeconomic challenges should be targeted with more general information, specific vaccine education and more viable equal access to vaccines. The interaction analysis of area and individual socioeconomic condition highlighted that individual household income was more important for vaccination than the living area; whereas, the area socioeconomic condition was more important than educational level suggesting vaccine programmes should be made to target those with low household income and living in areas with major socioeconomic challenges. What is also clear is that there is a lower vaccine uptake in those born outside of Sweden, particularly booster dose vaccination and in individuals with origin in central and eastern Europe, the Middle East and Africa. These tendencies are to some extent exacerbated by living in areas with low socioeconomic conditions. Although the probability of vaccination, particularly the third dose, remains low in these groups, living in an area with better socioeconomic conditions increases the percentage vaccinated. Targeted interventions to these groups with prebooked vaccinations could be one way of establish-ing and maintaining sufficient vaccine uptake.

## Introduction

 In 2019, COVID-19 emerged and was later declared as a global health crisis. The publication of the first genome sequence of SARS-CoV-2 enabled a rapid vaccine development^1^ and a total of 10 vaccines, including mRNA vaccines, protein subunit vaccines, inactivated vaccines and viral vector vaccines, were approved by WHO,^2^ three of which were available in Sweden. These included Comirnaty (BNT162b2 mRNA, BioNTech-Pfizer), Spikevax (mRNA-1273, Moderna) and Vaxzevria (ChAdOx1 nCoV-19, Oxford-AstraZeneca). COVID-19 was initially associated with high mortality, especially in older people and in patients with comorbidities. Despite the emergence of less virulent SARS-CoV-2 variants, maximising vaccine uptake with multiple doses in the population was still of importance^3^,^4^ and achieving a high rate of vaccination in the entire population is critical to reduce hospital admissions and help healthcare systems recover.^5^ Vaccine hesitancy is substantial in certain populations and was reported as one of the top 10 threats to global health by the WHO in 2019.^6^ This hesitancy, despite differences in populations and target diseases, has been shown to be partly driven by concerns about government control, vaccine safety and vaccine effectiveness.^7^ In Sweden and the UK, the rates of other types of vaccination for a variety of diseases have been lower among certain ethnic groups^8^,^9^ and in areas of higher deprivation.^10^ This also appears to be similar for COVID-19 vaccination with lower vaccine uptake reported in several defined groups.^11^,^13^ A population study of adults ≥18 years in the UK, regarding the first-dose vaccination rates, showed lower uptake among males, minority ethnic groups, areas with higher deprivation and lower educational attainment.^5^ In Sweden, specifically, male sex, lower income, living alone and being born outside Sweden were all associated with a lower vaccine uptake^14^ as well as higher mortality from COVID-19.^15^ Marked differences in COVID-19 vaccination have also been noted specifically among older adults with a Swedish study reporting the lowest uptake in individuals born in low and middle-income countries (LMIC).^14^ Studies on COVID-19 from LMICs suggest that vaccine acceptance was driven by personal protection, while side effects were the primary cause for hesitancy.^16^ Booster vaccine uptake has been shown to be lower than initial vaccination with differences in individual socioeconomic factors including education and income.^17^ A first step is to enrol individuals into a vaccination strategy by accepting the initial vaccination, but it is also of great importance to ensure they remain in the programme for the necessary booster doses.^18^ The current understanding of the importance of individual sociodemographic factors vs contextual regional area surroundings in COVID-19 vaccine uptake is lacking, as is the potential driving factors that influence decisions to vaccinate or not on both the individual and contextual area level. This study aimed to investigate both individual and area, sociodemographic determinants of COVID-19 vaccine uptake among adults in a general population from Southern Sweden—first, comparing no vaccine with receiving at least two doses and then receiving the third dose (booster) with those who only had two doses.

## Materials and methods

### Study population

The study cohort included all adults (≥18 years) residing in Scania (Skåne) county, a socioeconomically and ethnically diverse region in Southern Sweden, on 27 December 2020 (baseline) when vaccinations first started. All COVID-19 vaccination doses in Sweden were free of charge for the entire population and implemented in four sequential stages depending on age and health status, which is described in detail in the online supplemental information. The study cohort was followed until 12 June 2022. Individuals who died during follow-up were censored on the date of death.

### Patient and public involvement

There was no patient or public involvement in the design of this study and they were not made aware publicly about the study being carried out as this design was using register data in Sweden where all individuals are linked to national registers using a unique identifier which is then anonymised for research purposes. However, there may be a possibility to make the public aware of these results in the future once published.

### Register linkage, Exposure and covariates

Linkage from different register sources was achieved by using the personal identification number assigned to all Swedish inhabitants at birth or when attaining a permit of residence.^19^ Data on sociodemographics and on health status were collected at baseline. Individual-level data on date of birth, sex, country of birth and marital status were obtained from the regional population register. The country of birth was thereafter categorised into three groups: i) Sweden, ii) high-income countries (HIC) which included countries in other Nordic, western European countries and other countries (USA, Canada, Australia, etc.), and iii) LMIC which included countries in central and eastern Europe, the Middle East and Africa. Educational level, disposable household income and employment status were obtained from the Longitudinal integration database for health insurance and labour market studies (LISA) at Statistics Sweden. Disposable household income per consumption unit was calculated by dividing the sum of the disposable income of all members of the family by the total consumption weight of the family.^20^ Information on comorbidities, diagnoses and medical procedures within inpatient and specialised outpatient care was obtained from health records of the Scania Region between 2016 and 2020. Data on COVID-19 infection history (positive PCR tests for SARS-CoV-2), vaccine doses, vaccination dates and type of vaccine received during follow-up were collected from the electronic system SMINet and the National Vaccination Register both kept at the Public Health Agency of Sweden (Folkhälsomyndigheten). Previous COVID-19 infection was defined in two ways: first, by using the date of the first positive COVID-19 test reported prior to baseline inclusion date when comparing unvaccinated with at least two doses and later, defined as the first positive COVID-19 test reported prior to receiving the second vaccine dose when comparing those with only two doses with those with three or more vaccine doses. Area-level data on socioeconomic condition, obtained from Statistics Sweden for each Regional Statistics area (RegSO), were also used. Sweden is divided into 3363 different RegSO areas (466 in Skåne county; population size n=551–14 307) and each individual was linked to a RegSO using the residential address at baseline. On the RegSO area level, we had access to a socioeconomic index which is the simple arithmetic mean of three indicators: share of people with low economic standards, proportion of people with tertiary education and proportion of people who have had financial assistance for at least 10 months and/or have been unemployed for longer than 6 months.^21^ The index takes values between 0% and 100% and the higher the value of the index, the worse the socioeconomic conditions in a RegSO. The socioeconomic index in each RegSO has been used by Statistics Sweden to define area types categorised as 1—area with major socioeconomic challenges (index 22.0 or above; maximum 45.3 in available data for Skåne county), 2—area with socioeconomic challenges (index range 16.0–21.9), 3—socio-economically mixed area (10.1–15.9) and 4— area with good socioeconomic conditions (4.3–10.0), 5—area with very good socioeconomic conditions (index 4.2 or below; minimum 2.1 in available data).

### Outcome

The study outcomes were reception of no doses, ≥2 doses or ≥3 doses of an approved COVID-19 vaccine (Pfizer-BioNTech, AstraZeneca or Moderna) among the study individuals still alive by the end of follow-up on 12 June 2022, the date used for the present cross-sectional analysis. Individuals who received only one dose of COVID-19 vaccine but did not complete the two-dose recommendation at that time were thus excluded from the analyses (n=17 567 individuals, 1.65% of the study population) as they could not be reliably classified as vaccine acceptant or hesitant.

### Statistical analysis

Descriptive characteristics were presented as means, SD and frequencies (%). Associations between sociodemographic factors and vaccine uptake were modelled with logistic regression and reported as OR. The regression models were first established using main effects only and then extended with a prespecified set of two-way interaction terms all entered simultaneously, where socioeconomic index at the area level was interacted with country of birth (here categorised as high vs LMIC) and the three individual-level socioeconomic indicators (disposable household income, employment status and education). Clustering at the area level was handled in the regression models by including a random intercept for each RegSO. The initial analysis compared those with no vaccine doses vs those with ≥2 doses. Following that those with ≥3 doses were compared with those who only had two doses, that is, modelling the likelihood of receiving at least one booster dose among the vaccinated. Average marginal effects of these individual and area factors were estimated to calculate the percentage vaccinated. Further, the percentage point difference in vaccination probability between sociodemographic variables set at defined levels in combination with other variables was estimated. Statistical analyses were conducted in Stata SE 14.2 (Stata Corp.). Vaccine uptake by RegSO was mapped using R packages, sf, tmap, tm_shape, ggplot2, tm_polygons and R V.4.2.0 (R Foundation for Statistical Computing Vienna, Austria). The study was approved by the Swedish Ethical Review Authority (dnr 2021–00059).

## Results

**Figure 1:**
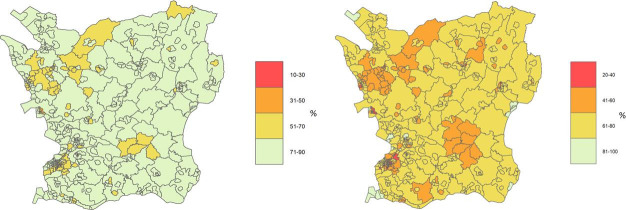
(A) Percentage of adult individuals vaccinated with two or more doses on 12 June 2022, stratified by Regional Statistics area (RegSO) in Skåne county, Sweden. (B) Percentage of adult individuals vaccinated with three or more doses on 12 June 2022, stratified by RegSO in Skåne county Sweden.

Baseline characteristics of the study population at the end of follow-up (n=1 064 548 after exclusion of 17 567 single-dose individuals) showed that 50% were female, mean age was 50 years (SD 19.1), 75% were born in Sweden, 60% were employed and 24% retired (table 1). There were 39% within the highest category (tertiary) of attainted level of education, 43% were married and 74% with no comorbid risk factors. Overall, 161 562 (15%) were unvaccinated, 245 685 (23%) received only two vaccine doses and 657 301 (62%) received the booster third dose. Differences across RegSO areas in vaccine uptake for the second and third doses are displayed in figure 1. On the area level, individuals living in areas with the lowest socioeconomic conditions were overrepresented among the unvaccinated (figure 1A,B).

**Table 1:** Baseline characteristics of the study population at the end of follow-up, stratified by vaccination status

Characteristics		Total	Unvaccinated	Two doses	At least three doses
N		1 064 548	161 562	245 685	657 301
Sex	Male	49.5	54.0	53.5	46.9
	Female	50.5	46.0	46.5	53.1
Previous infection (%)	No		95.5	94.0	89.2
	Yes		4.5	6.0	10.8
Age group (%)	<50	53.3	74.8	79.8	38.1
	50–64	22.7	16.3	15.3	27.0
	65–79	18.2	7.1	4.1	26.2
	≥80	5.8	1.8	0.8	8.6
Marital status (%)	Unmarried	39.7	53.0	55.1	30.6
	Married	42.7	31.6	33.4	48.9
	Divorced	12.9	13.5	10.2	13.8
	Widowed	4.7	1.9	1.3	6.7
Country of birth (%)	Sweden	74.5	48.6	63.6	84.9
	Other Nordic	2.6	3.8	2.0	2.5
	Western Europe	2.5	4.2	2.2	2.2
	Central and eastern Europe	8.9	22.8	11.2	4.6
	Middle East	6.5	13.1	13.2	2.5
	Africa	1.4	3.2	2.7	0.5
	Other	3.7	4.4	5.2	2.9
Education (%)	Primary	20.6	30.5	22.0	17.6
	Short secondary	18.8	17.1	13.8	21.1
	Long secondary	21.9	25.4	29.1	18.3
	Tertiary	38.8	27.1	35.1	43.0
Disposable household income (%)	≤1304	19.3	41.8	26.6	11.1
	1305–1748	19.6	19.7	19.6	19.6
	1749–2251	20.1	16.5	21.5	20.4
	2252–2954	20.4	13.4	19.4	22.5
	≥2955	20.6	8.7	12.9	26.5
Employment status (%)	Unemployed	12.2	30.9	17.8	5.6
	Employed	59.6	54.3	72.9	55.9
	Sickness absence	4.2	5.6	4.1	3.9
	Retirement	24.0	9.3	5.2	34.7
Number of risk factors (%)	No risks	73.6	83.4	83.7	67.5
	1 risk	17.6	12.7	12.6	20.6
	≥2 risks	8.8	3.9	3.7	11.9
Residential area type (%)	Area with major socioeconomic challenges	6.6	14.7	10.0	3.4
	Area with socioeconomic challenges	11.5	17.0	14.2	9.1
	Socioeconomically mixed area	24.2	27.4	25.9	22.8
	Area with good socioeconomic conditions	49.1	37.2	44.2	53.8
	Area with very good socioeconomic conditions	8.6	3.7	5.8	10.8

In the multivariable analysis, all investigated factors were associated with vaccine uptake, with similar patterns for both ≥2 doses and ≥3 doses (table 2). The percentage vaccinated, estimated as average marginal effects, was greater among females, older age groups, married individuals, those with higher attained educational level, being in employment, higher disposable household income and having more comorbid risk factors; whereas, the percentage vaccinated was lower with previous COVID-19 infection and being born outside of Sweden. Adjusted for individual-level factors, there was still a clear association between socioeconomic challenges at the area level and lower vaccine uptake for both ≥2 doses and ≥3 doses. Further grouping of country of birth into Sweden, HIC and LMIC (LMIC vs Swedish born) was associated with lower odds of vaccination with ≥2 doses vs 0 dose (OR 0.6, 95% CI 0.5 to 0.7) and (OR 0.3, 95% CI 0.2 to 0.3) for ≥3 doses vs remaining on only two (not in tables).

**Table 2:** ORs and percentage vaccinated in study population with at least two or at least three doses, estimated using multivariable logistic regression with main effects only

Characteristics		OR (95% CI)		Percentage vaccinated, average marginal effects (%)			
		At least two vs zero doses	At least three vs two doses	At least two doses	Percentage point difference	At least three doses	Percentage point difference
Sex	Male	Ref.	Ref.	84	2.0	60	4.0
	Female	1.2 (1.2 to 1.2)	1.3 (1.3–1.3)	86		64	
Age group	<50	Ref.	Ref.	82	11.0	52	33.0
	50–64	1.7 (1.6 to 1.7)	2.9 (2.8–2.9)	88		70	
	65–79	2.6 (2.4 to 2.8)	6.8 (6.4–7.1)	91		80	
	≥80	3.3 (3.0 to 3.5)	11.8 (10.8 12.8)	93		85	
Country of birth	Sweden	Ref.	Ref.	88		67	
	Other Nordic	0.5 (0.4 to 0.6)	0.6 (0.5–0.7)	78		56	
	Western Europe	0.6 (0.5 to 0.7)	0.9 (0.8–1.2)	78		58	
	Central and eastern Europe	0.4 (0.3 to 0.4)	0.3 (0.3–0.3)	66		39	
	Middle East	0.9 (0.8 to 1.0)	0.3 (0.2–0.3)	83		44	
	Africa	0.8 (0.6 to 0.9)	0.2 (0.2–0.3)	80		44	
	Other	1.8 (1.5 to 2.1)	0.5 (0.4–0.6)	87		60	
Civil status	Married	Ref.	Ref.	88	5.0	67	9.0
	Unmarried	0.6 (0.6 to 0.7)	0.6 (0.6–0.6)	88		65	
	Divorced	0.7 (0.7 to 0.7)	0.8 (0.8–0.8)	84		61	
	Widowed	1.0 (1.0 to 1.0)	0.8 (0.8–0.9)	83		58	
Prior infection	No	Ref.	Ref.	85	1.0	63	8.0
	Yes	1.2 (1.1 to 1.2)	0.8 (0.8–0.8)	86		55	
Comorbidities	None	Ref.	Ref.	84	6.0	60	10.0
	One	1.3 (1.3 to 1.4)	1.3 (1.3–1.3)	87		65	
	At least two	1.9 (1.8 to 1.9)	1.6 (1.5–1.6)	90		70	
Education	Primary	Ref.	Ref.	82	6.0	57	10.0
	Short secondary	1.2 (1.1 to 1.2)	1.4 (1.3–1.5)	83		60	
	Long secondary	1.1 (1.0 to 1.1)	1.2 (1.1–1.3)	83		58	
	Tertiary	1.5 (1.3 to 1.7)	1.7 (1.5–1.9)	88		67	
Employment status	Unemployed	Ref.	Ref.	78	9.0	53	11.0
	Employed	1.7 (1.6 to 1.8)	1.3 (1.2–1.4)	87		62	
	Sickness absence	1.7 (1.5 to 1.9)	2.0 (1.8–2.3)	84		64	
	Retired	1.6 (1.5 to 1.8)	1.7 (1.5–2.0)	86		67	
Household disposable income	Q1	Ref.	Ref.	79	11.0	52	18.0
	Q2	1.3 (1.2 to 1.4)	1.2 (1.1–1.3)	84		58	
	Q3	1.5 (1.4 to 1.7)	1.5 (1.4–1.7)	86		62	
	Q4	1.9 (1.7 to 2.1)	1.8 (1.7–2.0)	88		66	
	Q5	2.3 (1.9 to 2.7)	2.3 (2.0–2.7)	90		70	
Residential area type	Major challenge	Ref.	Ref.	77	11.0	1	14.0
	Challenge	1.6 (1.3 to 1.9)	1.4 (1.1–1.6)	83		60	
	Mixed	1.5 (1.3 to 1.7)	1.4 (1.2–1.6)	84		60	
	Good	1.7 (1.5 to 2.0)	1.7 (1.5–1.9)	86		63	
	Very good	2.0 (1.6 to 2.4)	2.0 (1.7–2.4)	88		67	

Percentage point difference, comparing reference category with highest category. Exeception, Mmarried compared tocompared with unmarried.

**a. Demographic characteristics and medical history. b. Individual-level and area-level socioeconomic characteristics.**

**Figure 2:**
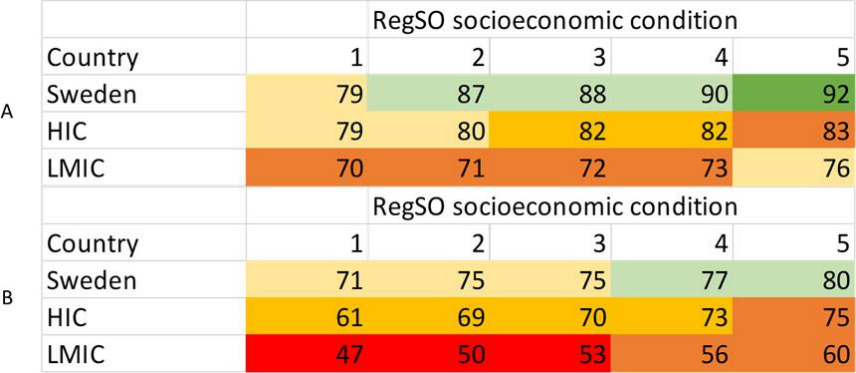
Average marginal effects, representing proportion receiving ≥2 doses (A) and proportion receiving booster dose (B) at a specific country category of birth and a specific level of socioeconomic condition on the regional level. RegSO socioeconomic condition; 1, areas with major socioeconomic challenges; 2, areas with socioeconomic challenges; 3, socioeconomically mixed areas; 4, areas with good socioeconomic conditions; 5, areas with very good socioeconomic conditions. High-income country (HIC). Low middle-income country (LMIC). RegSO, Regional Statistics area.

Interaction results for socioeconomic index at the area level in relation to individual-level factors are presented in figures2^4^ (country of birth, income and education) and online supplemental figure 1 (employment status). Absolute changes in percentage vaccinated across areas with different degree of socioeconomic challenges were largely similar across the individual-level factors, both for ≥2 doses and for the booster doses. However, for ≥2 doses uptake, a stronger association with the area level was noted for individuals born in Sweden than for those born abroad (figure 2). The percentage vaccinated was lowest among those born in central and eastern Europe, Middle East and Africa with the largest percentage point difference seen in those born in Africa for three or more doses, 20 percentage point difference (43% to 63%) comparing the lowest socioeconomic areas with the highest (online supplemental figure 2). This is highlighted by comparing born in Sweden to being born in a HIC or a LMIC. With greater area socioeconomic condition, the percentage vaccinated with ≥2 doses increased from 79%–92% among those born in Sweden; whereas, among those born in LMIC, it increased from 70%–76%. Considering three or more doses, the percentage vaccinated among those born in Sweden increased from 71%–80% with greater area socioeconomic condition and among those born in LMIC, the percentage vaccinated increased from 47%–60% (figure 2).

**Figure 3:**
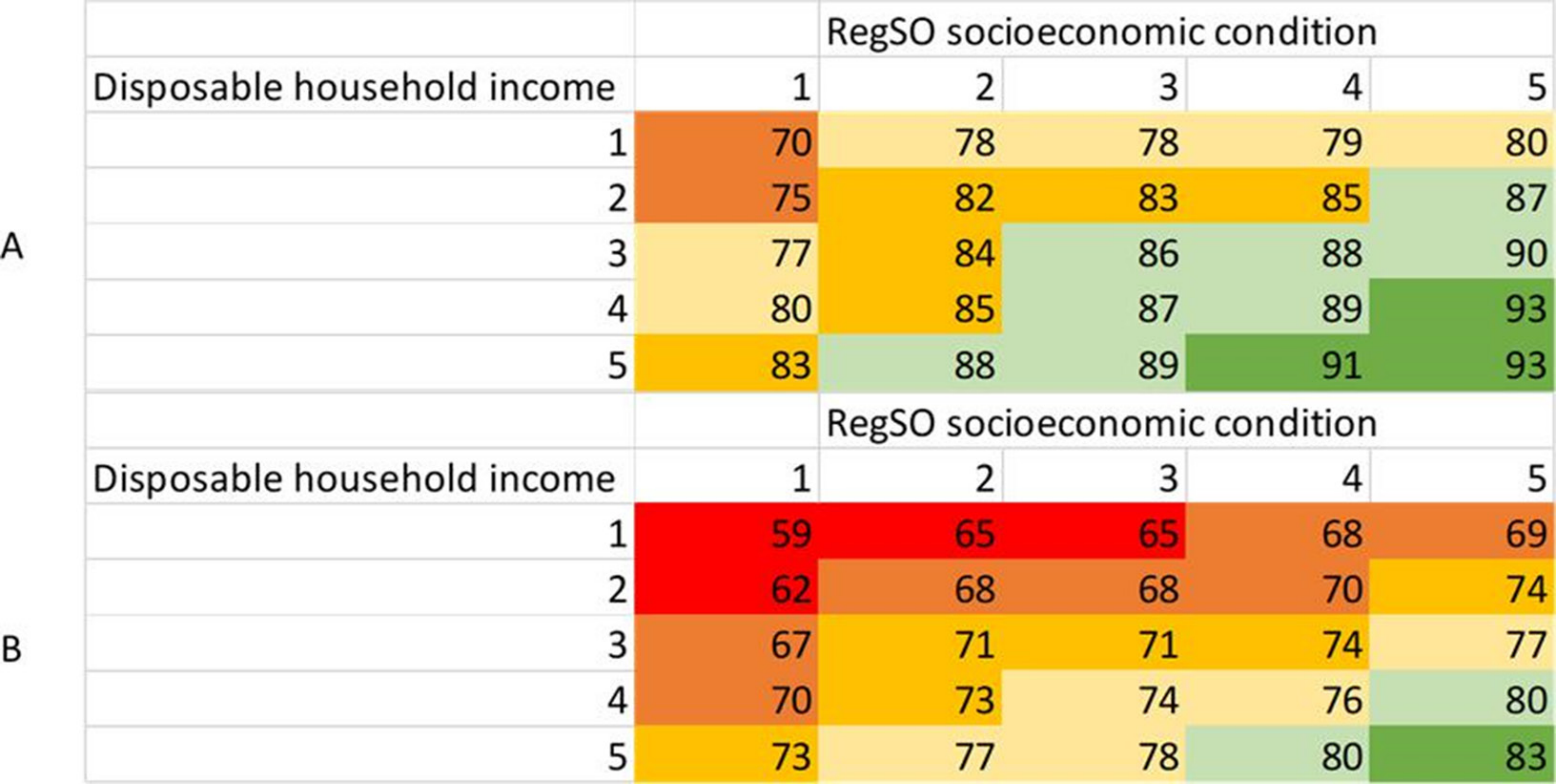
Average marginal effects, representing proportion receiving at ≥2 doses (A) and proportion receiving booster dose (B) at a specific quintile (Q1-Q5) of disposable household income and a specific level of socioeconomic condition on the regional level. RegSO socioeconomic condition; 1, areas with major socioeconomic challenges; 2, areas with socioeconomic challenges; 3, socioeconomically mixed areas; 4, areas with good socioeconomic conditions; 5, areas with very good socioeconomic conditions. RegSO, Regional Statistics area.

**Figure 4:**
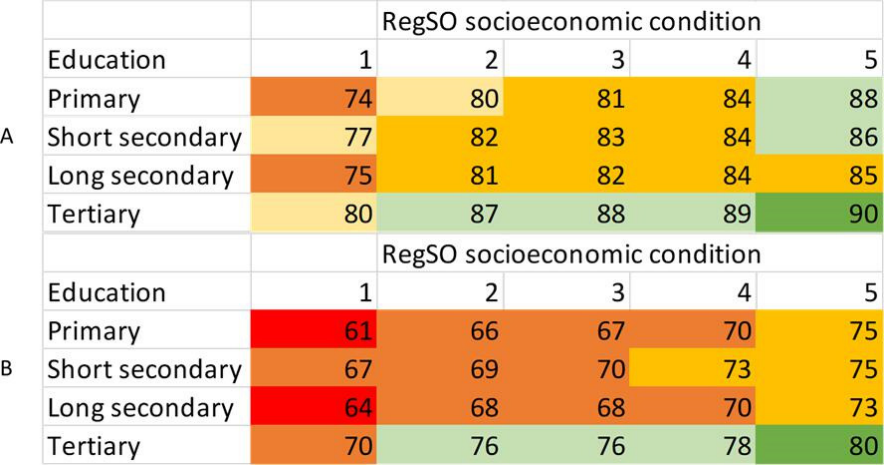
Average marginal effects, representing proportion receiving ≥2 doses (A) and proportion receiving booster dose (B) at a specific level of education and a specific level of socioeconomic condition on the regional level. RegSO socioeconomic condition; 1, areas with major socioeconomic challenges; 2, areas with socioeconomic challenges; 3, socioeconomically mixed areas; 4, areas with good socioeconomic conditions; 5, areas with very good socioeconomic conditions. RegSO, Regional Statistics area.

At the lowest level of disposable household income (Q1), the percentage vaccinated with at ≥2 doses increased from 70%–83% with increasing condition of socioeconomic area; whereas, at the highest level (Q5), the percentage vaccinated increased from 80%–93% (figure 3). This pattern was replicated for the third dose. At the lowest level of education (primary), the percentage vaccinated for ≥3 doses increased from 61%–75% with greater area socioeconomic condition; whereas, in the highest level of education (tertiary), the percentage vaccinated increased from 70%–80% (figure 4).

## Discussion

### Main findings

In this population-based study from Southern Sweden, our data showed consistent sociodemographic patterns both with respect to entering as well as remaining in the vaccination programme. There were clear gradients in the percentage vaccinated associated with sociodemographic factors both at the individual level as well as at the area level. There were notably low percentage vaccinated in some groups born outside of Sweden which should be a public health concern. We also found area-level associations independent of individual-level factors, with a clear gradient in greater percentage vaccinated with greater area socioeconomic conditions suggesting that the beliefs and decisions within the context where you live are important as well as your individual decision. The striking findings from this study show that remaining within the vaccination programme beyond just two doses is driven by the same differences in individual and area-level factors as the ones linked to get involved in the programme by accepting two doses which is of major importance for the vaccination strategies aiming at full population coverage. It is also evident that the lower percentage vaccinated for those born outside Sweden can be mitigated to some extent by area of residence. From the interaction analyses of area socioeconomic condition with individual-level household income and education, individual household income appears to be more important than the area for vaccination uptake; whereas, the area socioeconomic condition appears more important than individual education.

### Results in relation to previous studies

The previous research into COVID-19 vaccine uptake beyond single or second dose is limited but consistent with our findings. A recent study from Norway reported clear differences in vaccine uptake for two doses but also three or more booster doses regarding disposable household income and educational level.^17^ Further, a study performed in the US state of Massachusetts showed that first dose and subsequent booster dose was greater in areas with higher income and education and was lower in areas with a greater percentage of Black, Latino and indigenous adults.^22^ Additionally, among a specific population of healthcare workers in Wales, booster dose vaccine uptake was consistently lower among black healthcare workers, as well as those from deprived areas.^23^ These studies are in line with our results, with lower probability of booster vaccine among individuals with the lowest level of education, lowest disposable household income and those born outside of Sweden as well as in the areas with a low socioeconomic standing. Differences to consider between the studies are that in Massachusetts, in March 2022, a vaccine equity plan was put in place in attempt to encourage and inform the public particularly younger people about booster doses. Whereas, in Wales, the time period of 6 months post second dose was reduced to 3 months to encourage faster booster vaccination. In relation to these similar studies, what we have been able to add is the combination of individual-level and area-level socioeconomic status (SES) factors and the comparison of not just joining the vaccination programme but staying in the programme for booster doses. Among individual-level factors, we report both household disposable income and education were associated with vaccine uptake in line with a study from Canada, which demonstrated a clear gradient in vaccine uptake in relation to disposable household income^24^ and from Denmark, the lowest level of disposable income was associated with the lower odds of vaccination.^25^ In the same study from Denmark, the lowest level of education was also associated with lower odds of vaccination.^25^ In a study from Russia, the probability of vaccination for a person with high education was 24 percentage points higher than for a person with only general secondary education.^26^ We report that compared with individuals with the lowest level of education (primary), individuals with the highest level of education (tertiary) had a six percentage point greater and eight percentage point greater probability of being vaccinated with at least two or three or more doses, respectively, suggesting that the education disparities regarding vaccine uptake are lower in Sweden compared with Russia. Education has further been shown to be an important predictor of vaccine uptake in a US population study, with increasing odds of vaccination with higher levels of education.^27^ Similarly, we report the importance of education for vaccine uptake in our study; however, disposable household income may be an even stronger predictor. This notion may contrast a study from Norway which suggests education as the strongest predictor of SES.^28^

Considering country of birth, we reported a strong gradient with lower percentage vaccinated with two or three or more doses among individuals from all countries of birth compared with individuals born in Sweden. In England, all other ethnic groups had lower age-standardised rates of vaccination compared with the white British population. Black communities had the lowest rates, with 75% (74–75%) of black African and 66% (66–67%) of black Caribbean individuals having received at least one dose.^29^ We found that individuals born in Africa, compared with individuals born in Sweden, had the lowest probability of vaccination. In Denmark, descendants of non-western immigrants had lower odds of vaccination^25^ which is similar to the findings we report. We have been able to show that the effect of country of birth on the percentage vaccinated can be further affected by the area lived in.

### Potential mechanisms

The finding from our study that the difference in the second-dose uptake driven by socio demographic differences continues into the booster dose suggests that the same mechanisms operate for both. Those mechanisms could potentially be driven by both provider factors as well as target group factors.^30^ However, the design of this study makes it difficult to distinguish whether the difference in vaccine uptake depends on factors associated with service provider aspects including differences in accessibility (location of service points, opening hours, information given in appropriate language etc) and acceptability (social and cultural context of trust) or with target group or individual factors (ideologically driven vaccine hesitancy, low level of community commitment, etc), since we lack information regarding these factors. The results may in part also be explained by societal requirements for travel and activity, which also could be patterned by socioeconomic and demographic factors. Vaccine hesitancy has been identified among the target group factors^30^ and is reported to be higher among younger people, minority populations and socioeconomically vulnerable groups.^31^ Other factors include ethnicity, working status, religion and politics.^32^ There is evidence of various drivers behind vaccination hesitancy and vaccination unwillingness which may include inequities in health literacy, concern about own health and health of others, access and acceptance of side effects.^30^ These effects can be on the individual level but also on the area level with influence from family, friends, community members or public health policy makers.^33^ From a questionnaire study, the most given reasons to refuse vaccine were being against vaccines in general, concerns about safety/thinking that a vaccine produced in a rush is too dangerous, considering the vaccine useless because of the harmless nature of COVID-19 and a general lack of trust.^32^

### Strengths and limitations

The major strengths of our study are the large population data in a culturally diverse area of Sweden. Together with detailed individual register socioeconomic data, we also had access to area data that categorised regions into smaller populations living in proximity divided into categories based on their socioeconomic condition. This allowed us to identify not only individual socioeconomic status with variables such as disposable household income and education but also the economic situation in the particular area. This allows us to not only identify vulnerable groups who may need active campaigns to encourage vaccination but also vulnerable or deprived areas that have a low probability of vaccination. In terms of public health policy and vaccination programmes, this is key information for policy makers to make decisions on where to target campaigns, money and education in attempt to increase the vaccine uptake in these particular areas. One major strength of using the RegSO for the area data is that these defined regions are not affected by changes in postal code; therefore, any future analyses can be replicated.

A study limitation was that we were only able to report on those who received vaccination within Sweden. There are substantial differences between permanent and repeat immigrants in socioeconomic condition^34^ which as we have shown in this study is associated with vaccine uptake. Sweden and particularly Southern Sweden is a diverse multicultural setting and if individuals returned to their native countries or emigrated from Sweden, any further vaccinations would not appear in Swedish records; therefore, we may be underestimating some vaccine uptake. However, people travelling out from Sweden were often forced to have at least two doses to be let in the next country so we do not believe this would have a major impact on our findings. We also may not capture the true total number of previously infected individuals as not all COVID-19 cases appear in the registers which may bias results if prior infection is related to vaccination decision. Within this study, the possible marginal effects of any psychological predictors of COVID-19^35^ were unable to be analysed, such as concerns regarding unseen side effects,^7^ the rapid development, safety, commercial profiteering and effectiveness of the COVID-19 vaccine^36^ alongside fear regarding COVID-19 itself.^37^ However, these concerns and beliefs may well be associated with the individual-level and area-level sociodemographic factors that we in fact do study and are therefore captured by proxy in our population.

The interplay between socioeconomic deprivation and access to healthcare must also be considered as socioeconomic conditions are proportional to health outcomes and healthcare access^38^ which could impact ultimately on access to vaccination within economically vulnerable groups and places.^39^ However, this may have been less of a problem in the Swedish setting as the access into healthcare in Sweden is more equal economically than in many other countries^40^ and all vaccination doses were free for all. Perceived access and literacy in finding where to vaccinate or book at vaccinate may still differ by socioeconomic level.

### How can these results be used?

In an ideal system, vaccination should be equally informed free choice but our results show that this is not the case and disparities exist among certain groups and not even in Sweden with large general trust and trust in public institutions, particularly healthcare.^41^ If the information and access to vaccination were equal, then the uptake should be more random. This suggests that specific groups and specific areas with socioeconomic challenges should be targeted with more general information, specific vaccine education and more viable equal access to vaccination. The interaction analysis of area and individual socioeconomic condition highlighted that individual household income was more important for vaccination than living area characteristics; whereas, the area socioeconomic condition was more important than individual educational level suggesting that efforts should be made to target those with low household income and living in socioeconomically poor areas. What is also clear is that there is a lower vaccine uptake in those born outside of Sweden, particularly booster dose vaccination and in individuals with origin in central and eastern Europe, the Middle East and Africa. These tendencies are to some extent exacerbated by living in areas with low socioeconomic conditions. Although the probability for vaccination, particularly third dose, remains low in these groups, living in an area with better socioeconomic conditions increases the percentage vaccinated. Targeted interventions to these groups with prebooked vaccinations could be one way of establishing and maintaining sufficient vaccine uptake, as findings from a different region in Sweden suggest.^42^

## Conclusion

COVID-19 vaccine uptake was independently associated with higher sociodemographic classifications both at the individual level and area level. The percentage vaccinated increased with more affluent socioeconomic condition and concurrent increases in education and individual household income which appeared to be of greater importance for vaccination. This sociodemographic selection showed consistent patterns both with respect to entering (by obtaining at least two doses) and remaining (by obtaining at least one booster dose) in the vaccination programme. Further research is needed to understand why disparities exist in these groups and how they can best be addressed through public health policy and community engagement to increase initial and booster vaccination.

## supplementary material

10.1136/bmjph-2023-000437 online supplemental file 1

## Data Availability

Data are available upon reasonable request.
