# Supplementary material for: COVID-19 vaccine uptake in Skåne county, Sweden, in relation to individual-level and area-level sociodemographic factors: a register-based cross-sectional analysis
Source: BMJ Public Health. 2024 Mar 25;2(1):e000437. doi: 10.1136/bmjph-2023-000437 (PMC11812794; doi:10.1136/bmjph-2023-000437)
Supplement: online supplemental file 1 [file bmjph-2-1-s001.pdf]

Supplementary material

The vaccination program in Sweden started on 27th December 2020 and was implemented in four sequential stages. The first stage primarily focused on older adults in assisted living and residential care and health care workers or workers in the elderly care homes. The second stage then included all remaining adults ≥65 years, those with functional disabilities, and certain specific risk groups. By the 26th April 2021, the majority of regions within Sweden began stage three which included individuals aged 60−64 years, and various risk groups aged 18−59 years. The final stage included individuals aged 18−59 years who had not been part of phases 1−3 [(14)]. The third dose was first recommended for people with weakened immune systems 1stSeptember. By, 11th October booster doses were offered to those living in special housing then by 25th October, people aged 80 years and older were offered to be vaccinated at health care centers. Then on 15th November third dose vaccination was offered to those aged 65 years and older aswell as elderly care staff. Staff in inpatient care were offered a third dose starting 30th November. By 20th December third dose vaccination was offered to the rest of the population where at least six months have passed since dose two or five months for 65+.

Supplementary Figure 1. Average marginal effects, representing proportion receiving ≥2 doses (A) and proportion receiving booster dose (B) among the vaccinated at a specific employment status and a specific level of socioeconomic condition on the regional level. RegSO socioeconomic condition; 1 – areas with major socio-economic challenges, 2 – areas with socio-economic challenges, 3 – socio-economically mixed areas, 4 – areas with good socio-economic conditions, 5 – areas with very good socio-economic conditions

A

		RegSO socioeconomic condition			
Employment status	1	2	3	4	5
Unemployed	70	76	77	79	79
Employed	78	85	86	88	90
Sickness absenece	79	83	83	84	85
Retired	78	84	86	87	89

B

		RegSO socioeconomic condition			
Employment status	1	2	3	4	5
Unemployed	62	66	67	69	73
Employed	66	71	71	74	77
Sickness absenece	74	77	77	77	78
Retired	71	76	78	80	83

Supplementary Figure 2. Average marginal effects, representing proportion receiving ≥2 doses (A) and proportion receiving booster dose (B) among the vaccinated at a specific country of birth and a specific level of socioeconomic condition on the regional level. RegSO socioeconomic condition; 1 – areas with major socio-economic challenges, 2 – areas with socio-economic challenges, 3 – socio-economically mixed areas, 4 – areas with good socio-economic conditions, 5 – areas with very good socio-economic conditions.

A

	RegSO socioeconomic condition				
Country	1	2	3	4	5
Sweden	79	87	87	90	91
Other Nordic	67	76	78	79	82
Western Europe	71	71	79	79	82
Central and eastern Europe	61	64	65	67	70
Middle-East	77	81	82	84	86
Africa	75	78	79	80	83
Other	86	86	86	87	86

B

	RegSO socioeconomic condition				
Country	1	2	3	4	5
Sweden	71	75	75	77	80
Other Nordic	62	70	69	73	73
Western Europe	70	73	72	74	76
Central and eastern Europe	49	52	54	57	59
Middle-East	46	48	52	55	60
Africa	43	48	53	57	63
Other	59	68	69	72	75

Supplementary Table. Odds ratios and percentage vaccinated in study population with at least two or at least three doses, estimated using multivariable logistic regression with main effects only.

a. Demographic characteristics and medical history

Characteristics		OR (95% CI)	
		At least two vs. zero doses	At least three vs. two doses
Sex	Male	Ref.	Ref.
	Female	1.2 (1.2 - 1.2)	1.3 (1.3 - 1.3)
Age group	<50	Ref.	Ref.
	50-64	1.7 (1.7 - 1.8)	2.9 (2.8 - 2.9)
	65-79	2.7 (2.5 - 2.9)	6.8 (6.4 - 7.1)
	≥80	3.4 (3.2 - 3.7)	11.8 (10.8 12.8)
Country of birth	Sweden	Ref.	Ref.
	Other nordic	0.5 (0.4 - 0.6)	0.6 (0.5 - 0.7)
	Western Europe	0.6 (0.5 - 0.7)	0.9 (0.8 - 1.2)
	Central and Eastern Europe	0.4 (0.3 - 0.4)	0.3 (0.3 - 0.3)
	Middle-East	0.8 (0.8 – 0.9)	0.3 (0.2 - 0.3)
	Africa	0.8 (0.6 - 0.9)	0.2 (0.2 - 0.3)
	Other	1.7 (1.4 - 2.0)	0.5 (0.4 - 0.6)
Civil status	Married	Ref.	Ref.
	Unmarried	0.6 (0.6 - 0.6)	0.6 (0.6 - 0.6)
	Divorced	0.7 (0.7 - 0.7)	0.8 (0.8 - 0.8)
	Widowed	1.0 (1.0 - 1.0)	0.8 (0.8 - 0.9)
Prior infection	No	Ref.	Ref.
	Yes	1.1 (1.1 - 1.2)	0.8 (0.8 - 0.8)

Comorbidities	None	Ref.	Ref.
	One	1.3 (1.3 - 1.3)	1.3 (1.3 - 1.3)
	At least two	1.8 (1.7 - 1.8)	1.6 (1.5 – 1.6)

b. Individual- and area-level socioeconomic characteristics

Characteristics		OR (95% CI)	
		At least two vs. zero doses	At least three vs. two doses
Education	Primary	Ref.	Ref.
	Short secondary	1.2 (1.1 - 1.2)	1.4 (1.3 - 1.5)
	Long secondary	1.1 (1.0 - 1.1)	1.2 (1.1 - 1.3)
	Tertiary	1.5 (1.3 - 1.7)	1.7 (1.5 - 1.9)
Employment status	Unemployed	Ref.	Ref.
	Employed	1.6 (1.5 - 1.7)	1.3 (1.2 - 1.4)
	Sickness absence	1.7 (1.5 - 1.9)	2.0 (1.8 - 2.3)
	Retired	1.6 (1.4 - 1.7)	1.7 (1.5 - 2.0)
Household disposable income	Q1	Ref.	Ref.
	Q2	1.3 (1.2 - 1.4)	1.2 (1.1 - 1.3)
	Q3	1.5 (1.4 - 1.7)	1.5 (1.4 - 1.7)
	Q4	1.9 (1.7 - 2.1)	1.8 (1.7 - 2.0)
	Q5	2.3 (2.0 - 2.7)	2.3 (2.0 - 2.7)
Residential area type	Major challenge	Ref.	Ref.
	Challenge	1.5 (1.3 - 1.8)	1.4 (1.1 - 1.6)
	Mixed	1.5 (1.3 - 1.7)	1.4 (1.2 - 1.6)
	Good	1.7 (1.4 – 1.9)	1.7 (1.5 - 1.9)
	Very good	1.9 (1.6 – 2.3)	2.0 (1.7 - 2.4)
